# The Frank-Starling mechanism is not enough: blood volume expansion prominently decreases pulmonary O_2_ uptake

**DOI:** 10.1186/s40779-024-00546-3

**Published:** 2024-07-02

**Authors:** Mei-Han Guo, Candela Diaz-Canestro, David Montero

**Affiliations:** 1 Faculty of Medicine, Hong Kong University, Hong Kong, China; 2 Department of Medicine, School of Clinical Medicine, Hong Kong University, Hong Kong, China; 3 Libin Cardiovascular Institute of Alberta, University of Calgary, Calgary, T2N 4N1 Canada

**Keywords:** Blood volume expansion, Placebo-controlled, Cross-over, Pulmonary O_2_ uptake, Cardiac performance, Aerobic exercise capacity, Cardiorespiratory fitness

Dear Editor,

To date, the uncertainty remains whether the circulatory system of women and men can accommodate additional blood volume (BV) to enhance cardiac capacity [peak cardiac output (Q_peak_)] and aerobic capacity [peak O_2_ uptake (VO_2peak_)] [1–4]. In the general population, BV is typically below the optimal levels achieved through endurance training, suggesting that expanding BV may have clinical benefits [5]. This study aimed to determine whether: 1) moderate blood volume expansion (BVexp), similar in magnitude to that induced by endurance training interventions (+10% increase from each individual’s BV at baseline) [5], increases Q_peak_ and VO_2peak_ when assessed under a placebo (PBO)-controlled setting with a hydrostatically stable circulatory position; and 2) sex differences exist in the impact of BVexp on the healthy circulatory system of age- and physical activity-matched women and men. We hypothesized that BVexp (+10%) using a plasma volume (PV) expander (albumin 20%, CSL Behring, Germany) would result in proportional increments (+10%) in cardiac filling and Q_peak_, while not significantly affecting VO_2peak_ owing to the corresponding reduction in blood O_2_ carrying capacity. Forty healthy young women [*n* = 20, (26.2 ± 4.3) years] and men [*n* = 20, (27.2 ± 5.5) years] with an age range of 20–35 years were recruited via online advertisements after matching for age and physical activity level. For comparative purposes, women (*n* = 20) and men (*n* = 20) control groups who did not receive any type of intravenous infusion were also included (Additional file 1: Table S1). The exercise protocol involved a supine cycle ergometer integrated into a lower body pressure chamber, enabling a hydrostatically stable position for the (near)attainment of peak cardiac capacity [6]. Detailed rationale and methods are provided in Additional file 2.

Age and physical activity were well-matched between women and men (*P* ≥ 0.522) (Additional file 1: Table S1). In terms of cardiac variables at rest, the volumetric size of cardiac chambers, with or without normalization by body surface area (BSA), was found to be smaller in women compared to men (*P* ≤ 0.002, Additional file 1: Table S1). The effects of PBO and BVexp on resting hematological, cardiovascular, and hemodynamic variables are presented in Additional file 1: Table S2. Consistent with the study protocol, the amount of albumin infused during BVexp condition ranged from 476 to 618 ml per individual to achieve a 10% increase in PV and BV per kilogram of body weight. BVexp resulted in increased resting heart rate (HR) and left ventricular cardiac output (LV Q) in both sexes (*P* ≤ 0.007), whereas resting cardiac volumes, particularly in the left heart, were augmented only in men (*P* ≤ 0.017). Regarding arterial blood pressures and peripheral hemodynamics at rest, BVexp induced a minor increase (+3%) in mean arterial pressure (MAP) among men participants only (*P* = 0.015), whereas it led to a substantial decrease (–12 to –15%) in systemic vascular resistance (SVR) for both women and men (*P* = 0.004). For the blinding procedure, at the end of the 2nd visit, participants were asked about their guesses regarding which type of intravenous infusion they received (PBO or BVexp) during each visit. No participant was confident to conjecture. Additional file 1: Fig. S1 illustrates the effects of PBO and BVexp on cardiac volumes, HR, and LV Q from moderate exercise up until peak exercise. The absolute change (∆, BVexp minus PBO) in atrial and ventricular volumes was found to be larger in men compared to women (*P* for sex ≤ 0.045). In contrast, BVexp reduced HR throughout incremental exercise in men (*P* for condition < 0.001), but not in women (*P* for condition = 0.349), resulting in a similar ∆ in LV Q between sexes (*P* for sex = 0.795). The effects of PBO and BVexp on VO_2_ and arteriovenous O_2_ difference (a-vO_2diff_) are presented in Additional file 1: Figs. S2 and S3, respectively. Relative to PBO, BVexp decreased VO_2_ and a-vO_2diff_ during incremental exercise in women and men (*P* for condition < 0.001). The absolute reduction (∆) of VO_2_ and a-vO_2diff_ induced by BVexp was greater in men than in women (*P* for sex ≤ 0.001). Furthermore, SVR was reduced by BVexp during exercise in women and men (*P* < 0.05, Additional file 1: Fig. S3). When comparing with subjects not receiving any type of intravenous infusion (control), no difference was observed between the CTL and PBO groups (Additional file 1: Figs. S4-S6). Figure 1 illustrates the average percentage change elicited by BVexp relative to control (PBO) values in the main central and peripheral hemodynamic determinants of cardiorespiratory fitness. For comparative purposes, the presumed percentage change according to the initial hypothesis is concomitantly presented. LV filling (as represented by LVEDV) and LV SV at peak exercise (LVEDV_peak_ and LV SV_peak_) were approximately increased as per the presumed change (+10%) in women (+8.5% and +9.6% for LVEDV_peak_ and LV SV_peak_, respectively) and men (+8.3% and +8.6% for LVEDV_peak_ and LV SV_peak_, respectively). In contrast, HR_peak_, which was hypothesized to remain unchanged (0%), exhibits a slight decrease in women (–1.4%) and a significant decrease in men (–5.3%). Consequently, LV Q_peak_, expected to increase in proportion to BVexp (+10%), showed an increase of 8.3% in women but only a modest modification of 3.0% in men. The remaining Fick component, a-vO_2diff_ at peak exercise (a-vO_2diffpeak_), experienced substantial decreases exceeding the hypothesized decrement (–9%) both in women (–14.2%) and men (–12.5%). Finally, despite being anticipated to remain essentially unaltered (+1%), VO_2peak_ demonstrated considerable reductions among both women (–7.6%) and men (–9.6%).

**Fig. 1:**
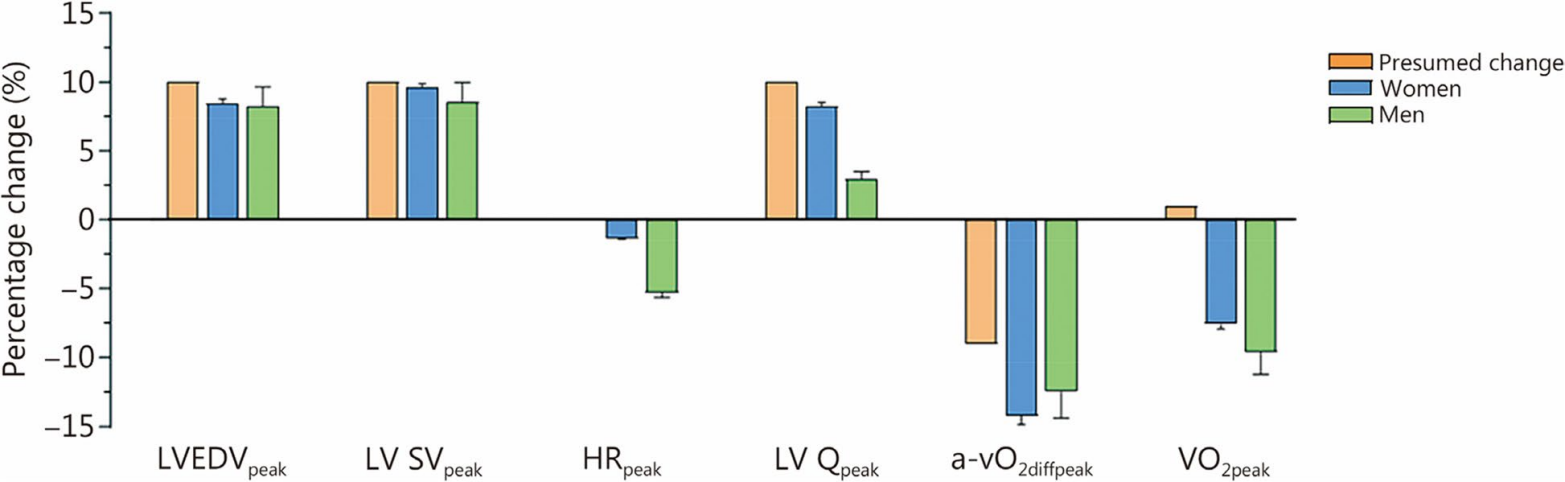
Presumed and observed percentage changes induced by blood volume expansion (BVexp) on main determinants of cardiac and aerobic capacities. Data are illustrated as the average percentage change: [(BVexp – PBO)/BVexp] × 100%, including the variability (SD) in observed variables. Conforming to the initial hypothesis, the presumed effects of BVexp (+10%) via albumin infusion comprised: no change in HR_peak_ (0%), proportional increments in left ventricular volumes and output (+10%), and nearly proportional decrements in a-vO_2diffpeak_ (–9%) resulting in no detectable change in VO_2peak_ (+1%). a-vO_2diffpeak_ arteriovenous O_2_ difference at peak exercise, HR_peak_ peak heart rate, LVEDV_peak_ left ventricular end-diastolic volume at peak exercise, LV Q_peak_ left ventricular cardiac output at peak exercise, LV SV_peak_ left ventricular stroke volume at peak exercise, PBO placebo, VO_2peak_ O_2_ uptake at peak exercise

To the best of our knowledge, no previous investigation had strictly controlled and maintained the optimal body position to preserve intravascular fluids throughout the entire duration of intravenous infusion until the completion of the incremental exercise test, as conducted in this present study (Additional file 2). When BV is acutely increased (+10%) via a PV expander (albumin infusion), while simultaneously controlling body position to maintain intravascular volumes, pulmonary VO_2_ remarkably decreased in women and men, without any discernible sex difference. These effects are primarily attributed to a pronounced reduction of systemic O_2_ extraction during incremental exercise, accompanied by altered systemic peripheral resistance to blood flow induced by BVexp. Consequently, typical increases in BV (+10%) resulting from endurance training interventions alone cannot fully explain the integrated enhancement of circulatory and aerobic capacities observed in healthy individuals with normal blood volume levels unless additional adaptations to endurance training and/or hypervolemia are present. Therefore, for the vast majority of individuals within the human population, interventions solely targeting modifications in circulatory system volume, a variable that can be readily adjusted, may not lead to functional improvements in aerobic capacity unless supplementary adaptations facilitate it.

In conclusion, this study demonstrates that the isolated expansion of BV up to the hypervolemic level typically induced by endurance training leads to significant alterations in peripheral circulatory function and reduced pulmonary VO_2_ in both women and men.

### Supplementary Information

**Additional file 1: Table S1 **General characteristics and body composition (*n* = 20). **Table S2** Effect of placebo (PBO) and blood volume expansion (BVexp) on hematological, cardiovascular, and hemodynamic variables at rest (mean ± SD). **Fig. S1** Effect of placebo (PBO) and blood volume expansion (BVexp) on cardiac volumes and output during moderate to peak exercise. **Fig. S2** Effect of placebo (PBO) and blood volume expansion (BVexp) on O_2_ uptake (VO_2_) during moderate to peak exercise. **Fig. S3** Effect of placebo (PBO) and blood volume expansion (BVexp) on arteriovenous O_2_ difference (a-vO_2diff_) during moderate to peak exercise, and arterial blood pressures (SAP_AT_, MAP_AT_) and systemic vascular resistance (SVR_AT_) at the anaerobic threshold (AT). **Fig. S4** Comparison of placebo (PBO) and control (CTL) groups regarding cardiac volumes and output during moderate to peak exercise. **Fig. S5** Comparison of placebo (PBO) and control (CTL) groups regarding O_2_ uptake (VO_2_) during moderate to peak exercise. **Fig. S6** Comparison of placebo (PBO) and control (CTL) groups regarding arteriovenous O_2_ difference (a-vO_2diff_) during moderate to peak exercise.

**Additional file 2:** **Detailed background, materials and methods, results, and discussion**.

## Data Availability

All data associated with this study will be available upon reasonable request to the corresponding author.

## Abbreviations

a-vO_2diff_: Arteriovenous O_2_ difference
BSA: Body surface area
BV: Blood volume
BVexp: Blood volume expansion
HR: Heart rate
PBO: Placebo
PV: Plasma volume
Q: Cardiac output
Q_peak_: Peak cardiac output
SVR: Systemic vascular resistance
VO_2_: O_2_ uptake
VO_2peak_: Peak O_2_ uptake
