# Supplementary material for: The Frank-Starling mechanism is not enough: blood volume expansion prominently decreases pulmonary O2 uptake
Source: Mil Med Res. 2024 Jul 2;11:43. doi: 10.1186/s40779-024-00546-3 (PMC11218401; doi:10.1186/s40779-024-00546-3)
Supplement: Supplementary file 1 — Additional file 1: Table S1 General characteristics and body composition (n = 20). Table S2 Effect of placebo (PBO) and blood volume expansion (BVexp) on hematological, cardiovascular, and hemodynamic variables at rest (mean ± SD). Fig. S1 Effect of placebo (PBO) and blood volume expansion (BVexp) on cardiac volumes and output during moderate to peak exercise. Fig. S2 Effect of placebo (PBO) and blood volume expansion (BVexp) on O2 uptake (VO2) during moderate to peak exercise. Fig. S3 Effect of placebo (PBO) and blood volume expansion (BVexp) on arteriovenous O2 difference (a-vO2diff) during moderate to peak exercise, and arterial blood pressures (SAPAT, MAPAT) and systemic vascular resistance (SVRAT) at the anaerobic threshold (AT). Fig. S4 Comparison of placebo (PBO) and control (CTL) groups regarding cardiac volumes and output during moderate to peak exercise. Fig. S5 Comparison of placebo (PBO) and control (CTL) groups regarding O2 uptake (VO2) during moderate to peak exercise. Fig. S6 Comparison of placebo (PBO) and control (CTL) groups regarding arteriovenous O2 difference (a-vO2diff) during moderate to peak exercise. [file 40779_2024_546_MOESM1_ESM.pdf]

Table S1 General characteristics and body composition

**Items**	**Experimental groups (n = 40)**		**Control groups (n = 40)**	
	**Women (n = 20)**	**Men (n = 20)**	**Women (n = 20)**	**Men (n = 20)**
Age (years, mean ± SD)	26.2 ± 4.3	27.2 ± 5.5	24.8 ± 4.1	24.6 ± 4.5
Height (cm, mean ± SD)	162.6 ± 7.4	172.4 ± 6.3†	163.4 ± 5.9	176.4 ± 7.3†
Weight (kg, mean ± SD)	54.1 ± 8.6	65.6 ± 7.9†	56.7 ± 8.5	70.0 ± 9.3†
BMI (kg/m^2, mean ± SD)	20.4 ± 2.3	22.0 ± 1.9†	21.1 ± 2.3	22.5 ± 2.1
BSA (m^2, mean ± SD)	1.57 ± 0.15	1.78 ± 0.13†	1.60 ± 0.10	1.90 ± 0.10†
MVPA (h/week, mean ± SD)	4.9 ± 3.8	5.5 ± 2.7	4.5 ± 4.7	5.0 ± 4.2
MVPA-END (h/week, mean ± SD)	4.1 ± 3.5	4.6 ± 2.6	3.5 ± 3.7	3.3 ± 3.6
Smoking [*n*(%)]	0	0	0	0
Body composition (mean ± SD)				
BMC (kg)	1.99 ± 0.25	2.50 ± 0.30†	2.04 ± 0.24	2.64 ± 0.40†
LBM (kg)	37.7 ± 6.1	52.5 ± 6.8†	40.4 ± 6.0	55.4 ± 7.3†
Absolute fat (kg)	15.3 ± 3.3	11.7 ± 2.5†	15.5 ± 4.8	13.4 ± 3.5
Relative fat (%)	27.8 ± 3.7	17.6 ± 3.1†	26.3 ± 5.8	18.6 ± 3.4†

Control groups were not subjected to any type of intravenous infusion. $^{\dagger}P < 0.05$, men vs. women. Data obtained in the reference testing (PBO condition) sessions are presented. Baseline data (before infusion) did not differ between testing sessions. *BMC* bone mineral content, *BMI* body mass index, *BSA* body surface area, *LBM* lean body mass, *MVPA* total moderate-to-vigorous physical activity, *MVPA-END* moderate-to-vigorous physical comprising endurance exercise, *PBO* placebo

Table S2 Effect of placebo (PBO) and blood volume expansion (BVexp) on hematological, cardiovascular, and hemodynamic variables at rest (mean ± SD)

Variables	PBO				BVexp			
	Baseline		Δ		Baseline		Δ	
	Women (n = 20)	Men (n = 20)	Women (n = 20)	Men (n = 20)	Women (n = 20)	Men (n = 20)	Women (n = 20)	Men (n = 20)
Blood								
Hb (g/dl)	12.3 ± 1.1	14.7 ± 1.0†	-0.3 ± 0.4*	-0.4 ± 0.5*	12.4 ± 1.1	14.9 ± 1.3†	-2.1 ± 0.6*	-2.6 ± 0.4*†
Hct (%)	37.7 ± 3.2	45.1 ± 3.0†	-1.1 ± 1.4*	-1.2 ± 1.4*	38.2 ± 3.4	45.7 ± 4.0†	-6.2 ± 1.7*	-7.7 ± 1.2*†
BV (ml/kg)[a]	90.4 ± 10.3	95.4 ± 11.8	+0.1 ± 0.0*	+0.1 ± 0.0*	88.9 ± 11.2	95.9 ± 14.0	+8.9 ± 1.1*	+9.5 ± 1.3*
PV (ml/kg)[a]	59.4 ± 7.6	56.2 ± 7.5	+0.1 ± 0.0*	+0.1 ± 0.0*	58.0 ± 8.6	56.7 ± 10.1	+8.9 ± 1.1*	+9.5 ± 1.3*
RBCV (ml/kg)[b]	31.0 ± 4.2	39.2 ± 5.4†	-	-	30.9 ± 4.0	39.2 ± 5.5†	-	-
Heart								
RA (ml/m^2)	14.2 ±2.4	18.3 ± 5.7†	+0.6 ± 2.0	-0.3 ± 1.9	14.5 ± 2.6	18.7 ± 4.7†	+0.8 ± 2.3	+1.1 ± 2.9
LA (ml/m^2)	16.3 ±3.6	20.1 ± 3.8†	+0.7 ± 2.8	-0.1 ± 3.5	15.4 ± 3.4	20.1 ± 4.8†	+0.5 ± 2.7	+2.1 ± 1.9*†
LVEDV (ml/m^2)	68.0 ± 7.3	79.2 ± 10.7†	-2.0 ± 5.5	+0.8 ± 4.5	64.8 ± 8.0	79.7 ± 11.5†	+2.8 ± 9.4	+7.4 ± 12.8*
LV SV (ml/m^2)	54.7 ± 5.6	62.7 ± 8.3†	-1.4 ± 5.6	+0.2 ± 3.9	52.8 ± 5.8	62.9 ± 9.2†	+2.2 ± 7.0	+7.7 ± 10.2*
HR (bpm)	57.8 ± 10.7	56.4 ± 6.9	+0.6 ±7.0	-2.7 ± 4.2*	60.8 ± 7.3	56.8 ± 7.9	+8.4 ± 9.7*	+4.9 ± 4.2*
LV Q [L/(min·m^2)]	3.1 ± 0.8	3.4 ± 0.7	-0.0 ± 0.7	-0.2 ± 0.4	3.2 ± 0.5	3.6 ± 0.7†	+0.6 ± 0.7*	+0.8 ± 1.1*
LV dia FR [ml/(s·m^2)]	97.8 ± 29.3	101.8 ± 24.3	-8.0 ± 25.8	-7.7 ± 18.4	94.5 ± 31.3	103.0 ± 20.1	+38.8 ± 35.0*	+28.7 ± 45.0*
LV sys ER [ml/(s·m^2)]	176.1 ± 22.2	210.9 ± 43.5†	-2.7 ± 25.6	-15.2 ± 22.7*	174.0 ± 21.1	204.5 ± 37.7†	+2.4 ± 26.2	+16.2 ± 42.1
Vascular								
SAP (mmHg)	100.7 ± 6.9	114.9 ± 6.8†	-0.5 ± 2.9	-0.6 ± 3.8	103.6 ± 9.4	112.7 ± 6.8†	+0.2 ± 4.1	+1.8 ± 5.5
DAP (mmHg)	67.6 ± 5.1	72.1 ± 6.9†	+0.5 ± 3.9	+0.8 ± 4.7	68.5 ± 6.9	71.2 ± 6.5	+0.1 ± 2.9	+2.1 ± 5.7
MAP (mmHg)	78.6 ± 4.9	86.3 ± 6.1†	+0.1 ± 3.4	+0.4 ± 4.0	79.6 ± 6.6	83.9 ± 5.2†	+1.1 ± 2.8	+2.9 ± 4.8*
SVR [dyn/(s·cm^5)]	1316 ± 247	1145 ± 230†	+40 ± 250	+63 ± 131*	1290 ± 154	1108 ± 256†	-162 ± 225*	-162 ± 219*

$^{*}P < 0.05$, post-infusion vs. baseline, within-sex in each condition (PBO, BVexp); $^{\dagger}P < 0.05$, men vs. women, referred to baseline or Δ in each condition (PBO, BVexp). [a]ΔBV and ΔPV were determined according to the amount of fluid infused. Hb concentration and Hct were not used to calculate ΔBV and ΔPV given that time per se modifies the

redistribution of PV within the circulatory system, augmenting the proportion of PV in the upper body in the supine body position, thereby reducing Hb concentration and Hct as measured in the upper limb, resulting in the overestimation of PV and BV. [b]RBCV was considered to be unaffected by intravenous infusions. Δ post-infusion minus baseline, *BV* blood volume, *DAP* diastolic arterial pressure, *Hb* hemoglobin concentration, *Hct* hematocrit, *HR* heart rate, *LA* left atrial volume, *LV dia FR* left ventricular diastolic filling rate, *LVEDV* left ventricular end-diastolic volume, *LV Q* left ventricular cardiac output, *LV SV* left ventricular stroke volume, *LV sys ER* left ventricular systolic emptying rate, *MAP* mean arterial pressure, *PV* plasma volume, *RA* right atrial volume, *RBCV* red blood cell volume, *SAP* systolic arterial pressure, *SVR* systemic vascular resistance to blood flow

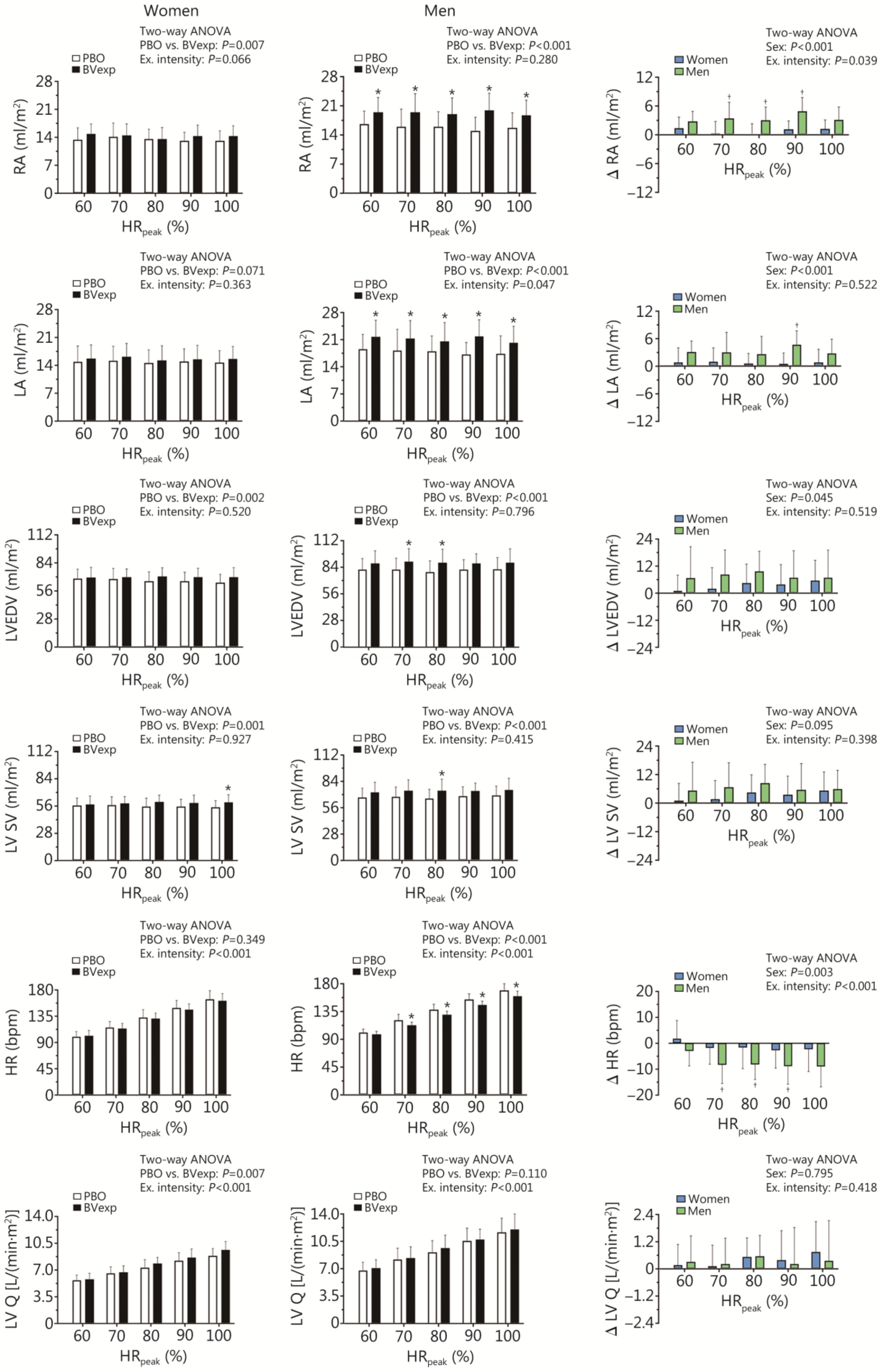
Women
Men
Two-way ANOVA
PBO vs. BVexp: P=0.007
Ex. intensity: P=0.066
PBO
BVexp
RA (ml/m²)
HRpeak (%)
Two-way ANOVA
PBO vs. BVexp: P<0.001
Ex. intensity: P=0.280
Two-way ANOVA
Sex: P<0.001
Ex. intensity: P=0.039
Women
Men
Δ RA (ml/m²)
Two-way ANOVA
PBO vs. BVexp: P=0.071
Ex. intensity: P=0.363
LA (ml/m²)
Two-way ANOVA
PBO vs. BVexp: P<0.001
Ex. intensity: P=0.047
Two-way ANOVA
Sex: P<0.001
Ex. intensity: P=0.522
Δ LA (ml/m²)
Two-way ANOVA
PBO vs. BVexp: P=0.002
Ex. intensity: P=0.520
LVEDV (ml/m²)
Two-way ANOVA
PBO vs. BVexp: P<0.001
Ex. intensity: P=0.796
Two-way ANOVA
Sex: P=0.045
Ex. intensity: P=0.519
Δ LVEDV (ml/m²)
Two-way ANOVA
PBO vs. BVexp: P=0.001
Ex. intensity: P=0.927
LV SV (ml/m²)
Two-way ANOVA
PBO vs. BVexp: P<0.001
Ex. intensity: P=0.415
Two-way ANOVA
Sex: P=0.095
Ex. intensity: P=0.398
Δ LV SV (ml/m²)
Two-way ANOVA
PBO vs. BVexp: P=0.349
Ex. intensity: P<0.001
HR (bpm)
Two-way ANOVA
PBO vs. BVexp: P<0.001
Ex. intensity: P<0.001
Two-way ANOVA
Sex: P=0.003
Ex. intensity: P<0.001
Δ HR (bpm)
Two-way ANOVA
PBO vs. BVexp: P=0.007
Ex. intensity: P<0.001
LV Q [L/(min·m²)]
Two-way ANOVA
PBO vs. BVexp: P=0.110
Ex. intensity: P<0.001
Two-way ANOVA
Sex: P=0.795
Ex. intensity: P=0.418
Δ LV Q [L/(min·m²)]

Fig. S1 Effect of placebo (PBO) and blood volume expansion (BVexp) on cardiac volumes and output during moderate to peak exercise. Data are illustrated as mean ± SD. $^{*}P < 0.05$, BVexp vs. PBO, within-sex at a specific exercise intensity in each condition (PBO, BVexp); $^{\dagger}P < 0.05$, men vs. women, referred to Δ at a specific exercise intensity. Δ BVexp minus PBO, HR heart rate, HR_{peak} peak heart rate, RA right atrial volume, LA left atrial volume, LVEDV left ventricular end-diastolic volume, LV Q left ventricular cardiac output, LV SV left ventricular stroke volume, Ex. exercise

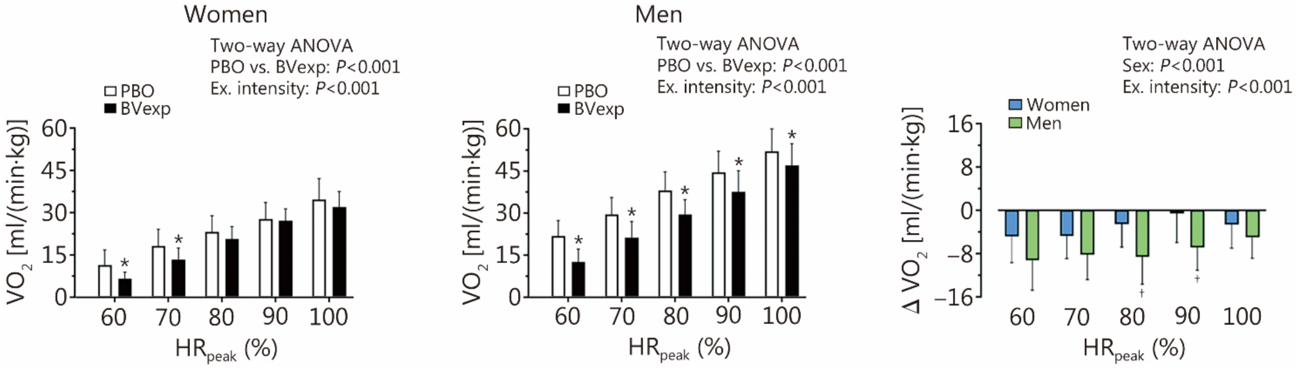

Fig. S2 Effect of placebo (PBO) and blood volume expansion (BVexp) on O_2 uptake (VO_2) during moderate to peak exercise. Data are illustrated as mean ± SD. $^{*}P < 0.05$, BVexp vs. PBO, within-sex at a specific exercise intensity in each condition (PBO, BVexp); $^{\dagger}P < 0.05$, men vs. women, referred to Δ at a specific exercise intensity. Δ BVexp minus PBO, HR_{peak} peak heart rate, Ex. exercise

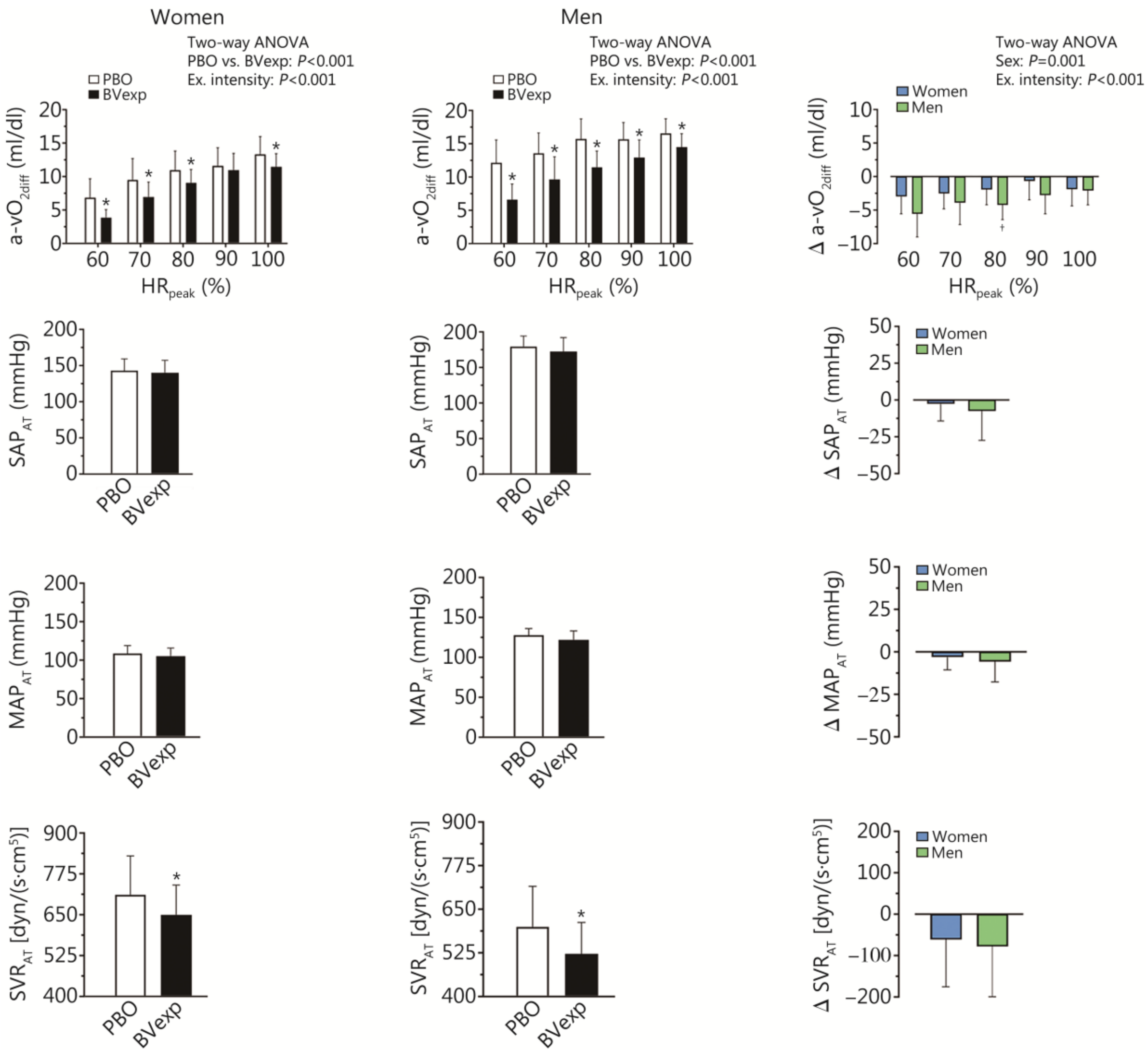

Fig. S3 Effect of placebo (PBO) and blood volume expansion (BVexp) on arteriovenous O_2 difference ($a\text{-}vO_{2diff}$) during moderate to peak exercise, and arterial blood pressures (SAP_{AT}, MAP_{AT}) and systemic vascular resistance (SVR_{AT}) at the anaerobic threshold (AT). Data are illustrated as mean ± SD. Average percentage of peak heart rate (HR_{peak}) at the anaerobic threshold: 87 – 89% in both conditions (PBO, BVexp) in women and men. $^{*}P < 0.05$, BVexp vs. PBO, within-sex at a specific exercise intensity in each condition (PBO, BVexp); $^{\dagger}P < 0.05$, men vs. women, referred to Δ at a specific exercise intensity. Δ BVexp minus PBO, MAP_{AT} mean arterial pressure at the anaerobic threshold, SAP_{AT} systolic arterial pressure at the anaerobic threshold, SVR_{AT} systemic vascular resistance at the anaerobic threshold, Ex. exercise

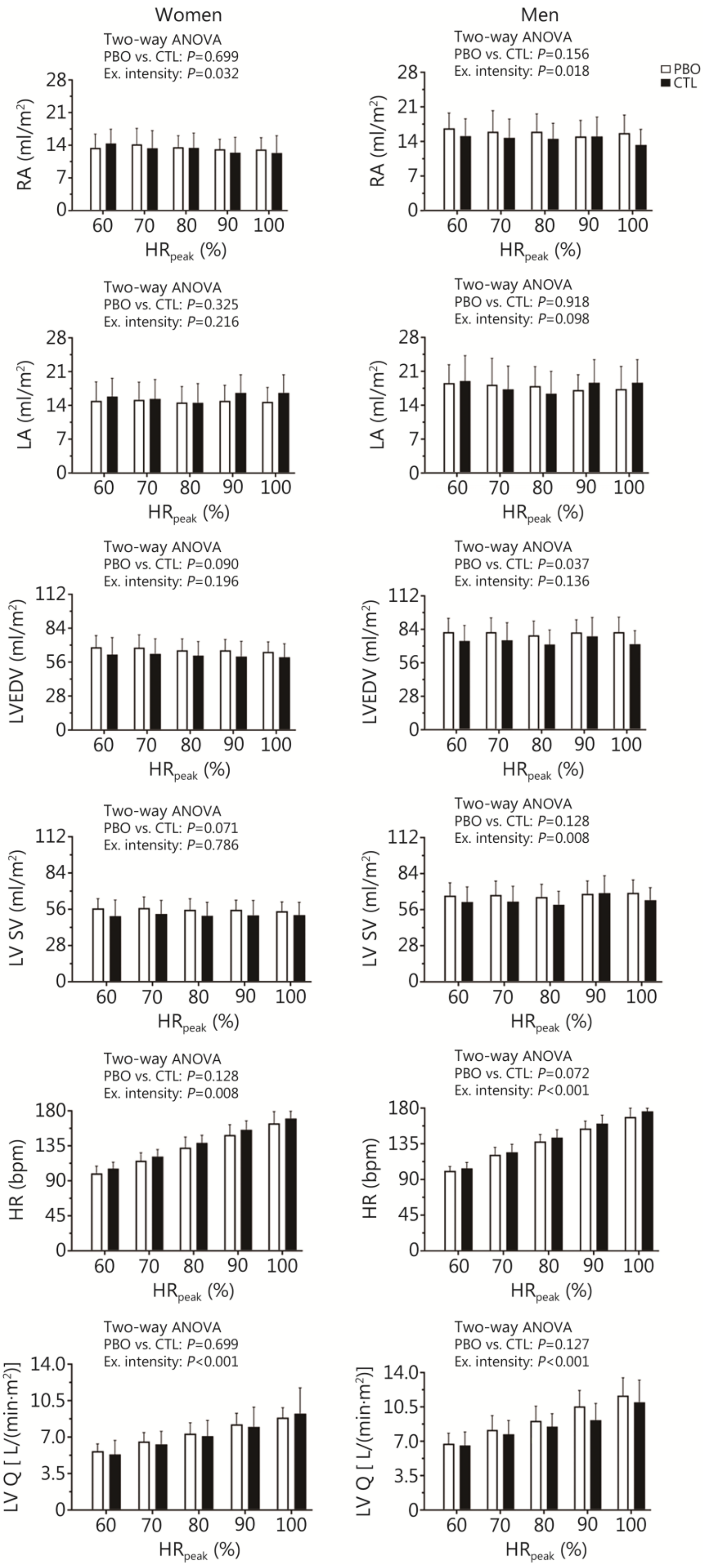
Women
Men
□ PBO
■ CTL
Two-way ANOVA
PBO vs. CTL: P=0.699
Ex. intensity: P=0.032
RA (ml/m2)
Two-way ANOVA
PBO vs. CTL: P=0.156
Ex. intensity: P=0.018
Two-way ANOVA
PBO vs. CTL: P=0.325
Ex. intensity: P=0.216
LA (ml/m2)
Two-way ANOVA
PBO vs. CTL: P=0.918
Ex. intensity: P=0.098
Two-way ANOVA
PBO vs. CTL: P=0.090
Ex. intensity: P=0.196
LVEDV (ml/m2)
Two-way ANOVA
PBO vs. CTL: P=0.037
Ex. intensity: P=0.136
Two-way ANOVA
PBO vs. CTL: P=0.071
Ex. intensity: P=0.786
LV SV (ml/m2)
Two-way ANOVA
PBO vs. CTL: P=0.128
Ex. intensity: P=0.008
Two-way ANOVA
PBO vs. CTL: P=0.128
Ex. intensity: P=0.008
HR (bpm)
Two-way ANOVA
PBO vs. CTL: P=0.072
Ex. intensity: P<0.001
Two-way ANOVA
PBO vs. CTL: P=0.699
Ex. intensity: P<0.001
LV Q [L/(min·m2)]
Two-way ANOVA
PBO vs. CTL: P=0.127
Ex. intensity: P<0.001
HRpeak (%)

Fig. S4 Comparison of placebo (PBO) and control (CTL) groups regarding cardiac volumes and output during moderate to peak exercise. Data are illustrated as mean ± SD. HR heart rate, HR_{peak} peak heart rate, RA right atrial volume, LA left atrial volume, LVEDV left ventricular end-diastolic volume, LV Q left ventricular cardiac output, LV SV left ventricular stroke volume, Ex. exercise

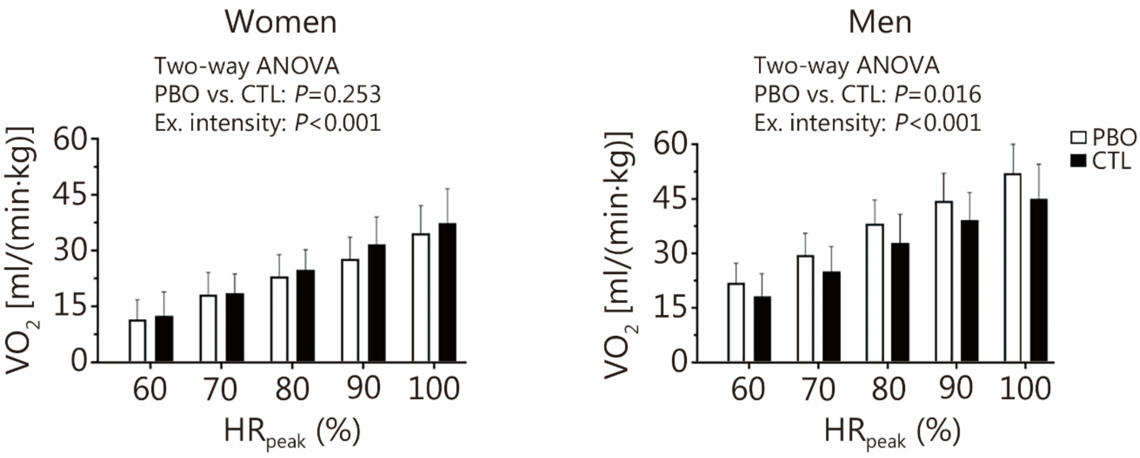

Fig. S5 Comparison of placebo (PBO) and control (CTL) groups regarding O_2 uptake (VO_2) during moderate to peak exercise. Data are illustrated as mean ± SD. HR_{peak} peak heart rate, Ex. exercise

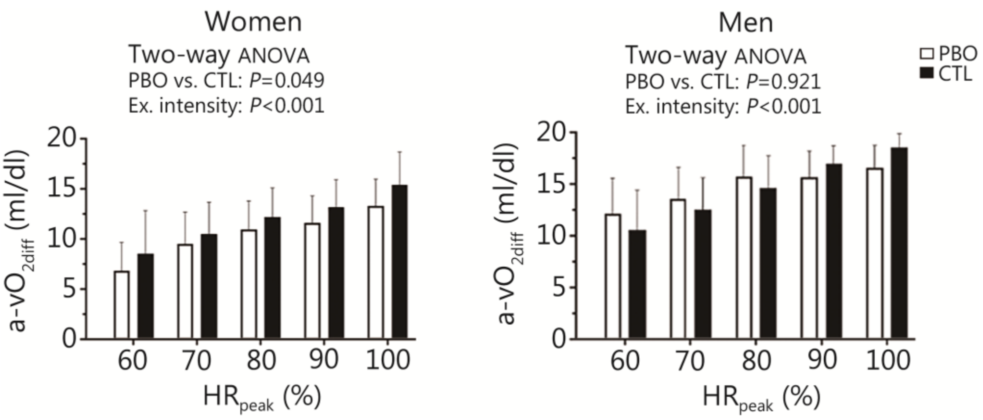

Fig. S6 Comparison of placebo (PBO) and control (CTL) groups regarding arteriovenous O_2 difference (a-vO_{2diff}) during moderate to peak exercise. Data are illustrated as mean ± SD. HR_{peak} peak heart rate, Ex. exercise
