# Supplementary material for: The Frank-Starling mechanism is not enough: blood volume expansion prominently decreases pulmonary O2 uptake
Source: Mil Med Res. 2024 Jul 2;11:43. doi: 10.1186/s40779-024-00546-3 (PMC11218401; doi:10.1186/s40779-024-00546-3)
Supplement: Supplementary file 2 — Additional file 2: Detailed background, materials and methods, results, and discussion. [file 40779_2024_546_MOESM2_ESM.pdf]

Background

It has been seventy-five years since the explicit recognition of the relationship between blood volume (BV) and cardiorespiratory fitness [1]. A substantial amount of observational and experimental evidence, mainly using blood withdrawal as an intervention, has been accumulated thus far, indicating a straightforward concept: greater BV corresponds to higher pumping capacity by a healthy heart [2-5]. Enhanced cardiac pumping capacity [henceforth referred to as "cardiac capacity" or "left ventricular (LV) peak cardiac output (Q_{peak})"] results in an increased potential for oxygen (O_2) delivery to the tissues and thereby higher peak O_2 uptake (VO_{2peak}), which is strongly associated with performance (endurance) and clinical outcomes such as all-cause mortality [6,7]. The precedent assertion assumes that blood O_2 carrying capacity, represented by hemoglobin (Hb) concentration, remains constant or at least is not significantly decreased when BV is increased. Indeed, the improvement in VO_{2peak} observed after blood reinfusion − a potent method of blood doping involving the reinfusion of red blood cells [8] − appears to be primarily attributed (94%) to the concurrent increase in Hb concentration [9]. However, from a cardiovascular and health perspective, an enhanced VO_{2peak} without an increase in cardiac capacity may have limited clinical relevance [10,11]. Using a car analogy, enhancing the horsepower of a car solely through higher fuel quality, without making any intrinsic modifications of the engine, may not result in substantial improvements.

The question of whether blood volume expansion (BVexp) per se, independent of blood O_2 carrying capacity and non-hematological factors, could improve cardiac capacity and VO_{2peak} has been extensively investigated by outstanding physiologists during the latter quarter of the 20th century [2,5,12-17]. These studies involved acute intravenous infusion (200 – 1000 ml) of a plasma volume (PV) expander comprising a complex branched polysaccharide (dextran) in healthy young individuals [2,5,12-17]. Overall findings from these pioneering investigations revealed that PV expansion increases cardiac capacity and VO_{2peak} in humans under two conditions: 1) when the infused volume does not exceed 400 ml and 2) when the individual does not initially exhibit hypervolemia commonly observed in highly trained endurance athletes [2,5,12-17]. In conclusion, it can be inferred that moderate PV infusion enhances cardiac filling and output to a higher extent than the resultant reduction in blood O_2 carrying capacity affects VO_{2peak} in a healthy but not fully expanded circulatory system.

The available evidence on this topic raises three important considerations. Firstly, it is worth noting that all studies have allowed participants to assume an upright position after intravenous infusion to assess peak exercise capacity [2,5,12-20]. However, the impact of gravity and hydrostatic pressure on PV cannot be ignored in this context. When standing upright, more than 500 ml of plasma (approximately 10% of BV) is redistributed out into the interstitial fluid of the lower limbs due to hydrostatic pressure- and time-dependent manner (at a rate of about 125 ml/min) [21,22]. This raises concerns regarding the accuracy and consistency of assessing cardiac and aerobic capacities during

these conditions. Secondly, it should be noted that the precise volume of infused volume relative to each individual's blood volume was not determined in previous studies. As a result, different individuals may have experienced varying volumetric stimuli (percentage change in BV) [2,5,12-20]. For sedentary untrained individuals with low BV, excessive infusion volumes may have been administered beyond optimal circulatory system filling. Conversely for endurance-trained athletes with high blood volumes, the same infusion volume might not have been sufficient enough to elicit detectable effects. Thirdly, the majority of previous studies did not blind the subjects to the experimental intervention [5,12-18]. Therefore, it remains necessary to conduct controlled experiments under stable hydrostatic conditions while ensuring definite volumetric stimuli strictly relative to the BV of each individual, to ascertain the effect of changes in BVexp on cardiac and aerobic capacities.

When previous studies were conducted, there was limited knowledge regarding the structural and functional sex dimorphism in cardiorespiratory physiology, coupled with a lesser emphasis on including women as participants [23-26]. Indeed, over a span of twenty-five years, only one of the aforementioned studies partially included women (n = 7) [17]. To date, the potential capacity of the female circulatory system to accommodate additional BV for improved cardiac capacity and VO_{2peak} remains largely unexplored. It is known, however, that women generally exhibit blunted cardiac enlargement (eccentric remodeling) in response to endurance training [24,27,28], despite their ability to significantly increase BV [29]. Moreover, a large population study indicates that women have stiffer hearts compared to men, which could contribute to restricting cardiac enlargement in females [30]. Therefore, it is possible that the sex-specific cardiac phenotype in women may not adapt to BVexp as effectively or to the same extent as in men. If this is indeed the case, lifestyle and pharmacological interventions aimed at improving cardiac and aerobic capacities for cardiovascular health benefits should not primarily focus on targeting BV in the female population.

Materials and methods

Study participants

Women [n = 20, 26.2 ± 4.3 (20 – 35) years] and men [n = 20, 27.2 ± 5.5 (20 – 35) years] matched by age and physical activity were recruited (until June 2023). Exercise training history and lifetime moderate-to-vigorous physical activity (MVPA), including specific endurance exercise details during the last 3 months prior to the study as previously described [31]. In addition, women (n = 20) and men (n = 20) matched by age, sex, and physical activity with the experimental groups were included (until June 2023). Inclusion criteria involved meeting health/clinical questionnaire requirements and passing resting echocardiography/electrocardiogram screening, without current medical symptoms or medication use while having no history of disease. The study was approved by the Institutional Review Board of the University of Hong Kong and the Hospital Authority West Cluster (UW 21-401) in

accordance with the Declaration of Helsinki. Prior to the start of the experiments, informed oral and written consents were obtained from the participants.

Study design

General design

Participants were required to attend our laboratory for testing on two occasions. The exercise protocol was identical in both sessions, except for the intravenous infusion condition [placebo (PBO) via saline infusion, BVexp via albumin infusion] which was blinded to the participants. Due to the potentially long half-life of albumin in the human body (up to 19 d), the order of testing sessions was not randomized (PBO on day 1 and BVexp on day 2) to prevent confounding carryover effects. The participants were instructed to avoid strenuous exercise, alcohol, and caffeine 24 h prior to testing, as well as to maintain their usual baseline activity and daily dietary habits throughout the study period. Testing sessions occurred at a consistent time of day for each participant with a gap of 2 – 7 d between sessions. All measurements were performed after a fasting period of at least 4 h to avoid postprandial hemodynamic alterations [32]. According to previous studies, the menstrual phase was noted but not fixed for testing as it does not affect the study outcomes [3,33]. The day of the menstrual cycle (for a 28-day normalized cycle) was found to be (11.7 ± 8.2) d and (13.1 ± 8.9) d for the 1st and 2nd testing sessions, respectively. Prior to testing, the participants completed general and clinical questionnaires encompassing disease history (including family medical history), physical activity history, diet/supplement intake history (in case of any reported food restriction, such as vegetarian diets), female-specific health information related to menstruation, blood donation history (if applicable), as well as detailed information regarding recent physical activity, food consumption, supplement usage, medication (if any) and fluid intake over the last 24 h. Thereafter, the participants were subjected to anthropometric measurements and body composition assessments before resting in the supine position for 15 min on the testing platform in order to stabilize cardiovascular and hematological variables.

Specific design

Hematological, cardiac, hemodynamic, and pulmonary measurements were performed in a supine position upon a horizontal platform 95 cm above the ground. During rest pre-infusion, rest post-infusion, and incremental exercise, participants' legs were either relaxed and extended (for resting measurements) or flexed with their feet fastened to the cycle ergometer (for exercise measurements), as detailed in the "Measurements" section. To ensure the blinding of participants to the intravenous infusion condition, a blackout curtain surrounded the platform except for a small incision allowing access to the left arm without revealing what was behind the curtain. The left arm – where intravenous infusion was performed, blood samples were taken and blood pressures were measured – was carefully placed and fixed during the whole testing period at the heart height level (approximately 90° abduction).

In this position, cannulation of the most prominent vein in the antecubital fossa of the left arm was cannulated using an Introcan Safety® IV Catheter (22G, B. Braun Medical Inc., Germany). Following blood sampling/gas analyses, and cardiac and hemodynamic measurements at rest (lasting a total of 15 min), the intravenous infusion started. In the PBO condition, 10 ml NaCl (BD 0.9% NaCl, BD, USA) was infused over a period of 50 min to match the expected duration based on previous experiments [32]. In the BVexp condition, human albumin (Albumin CSL 20%, CSL Behring, Germany) was administered intravenously over a period of (53 ± 11) min in order to induce a 10% increase of BV, taking into account the residual fluid in the infusion set (T-500, JMS, Singapore). In both (PBO, BVexp) conditions, ambient lighting was dimmed and instrumental music was played to facilitate relaxation during the infusion period and mask auditory cues for participants (e.g., the opening of albumin bottles). In addition, most participants engaged with their cell phones/tablets. After completion of the infusion, the same resting measurements were conducted as before the infusion (15 min). Subsequently, the feet of the participants were secured in an ergometer, an elastic belt was moderately tightened at the iliac crest and a face mask connected to a metabolic cart was adjusted to initiate the incremental exercise test along with its respective measurements described below.

Measurements

Cardiac volumes and hemodynamics

Apical four-chamber and two-chamber cine-loops were recorded and assessed offline via high-resolution ultrasound (M9, Mindray Medical, Hong Kong, China) during rest and at predetermined levels of exercise intensity according to peak heart rate (HR_{peak}) (60%, 70%, 80%, 90%, and 100% HR_{peak}) during the incremental exercise test (detailed in the next subsection). An electromagnetic cycle ergometer (KICKR Core, Wahoo, USA) was integrated into the horizontal platform inside a self-manufactured lower body pressure (LBP) chamber that allows moderate left-lateral tilting (set to 17° relative to the horizontal), facilitating precise cardiac imaging while cycling from rest until peak exercise. This setup has been established in previous studies [26,34,35]. The ambient pressure inside the LBP during exercise is maintained at –50 mmHg to induce the hemodynamic load characteristic of the upright position [36], which is necessary for achieving HR_{peak}, LV $Q_{peak,}$ and VO_{2peak} in humans [34,37,38]. In this respect, supine cycling exercise with an LBP of –50 mmHg is considered a model for upright exercise with regard to the central and peripheral circulation in skeletal muscle [37]. However, there may still be slight quantitative differences between exercise with an LBP of –50 mmHg and performing upright exercise regarding central factors such as HR_{peak} or peripheral factors like blood flow distribution [39]. Following the recommendations of the American Society of Echocardiography and the European Association of Cardiovascular Imaging, cardiac chamber quantification including left ventricular end-diastolic volume (LVEDV) and left ventricular end-

systolic volume (LVESV) was performed using the modified Simpson method (biplane method of disks) by tracing the endocardial border of the LV in apical four-chamber and two-chamber views at end-diastole and end-systole [40,41]. The difference between LVEDV and LVESV provided LV stroke volume (SV). LV cardiac output (LV Q) was calculated as the product of LV SV and HR. LV diastolic filling rate (LV dia FR) and LV systolic emptying rate (LV sys ER) were respectively calculated as LV SV divided by filling time or ejection time [16], determined from tissue Doppler imaging recordings in the septal wall adjacent to the mitral annulus. Right atrial (RA) and left atrial (LA) volumes were determined from the apical four-chamber view at end-systole, right before mitral valve opening. Due to the uncertain imaging quality, right ventricular dimensions were not analyzed given its morphologically complex chamber [42]. In our laboratory, echocardiographic (volumetric) measurements during incremental exercise have shown reproducibility with a within-subject coefficient of variation of ≤ 7% for atrial volumes (RA, LA), and ≤ 6% for LV volumes. To eliminate confounding effects related to body size, cardiac volumetric variables are commonly normalized by body surface area (BSA; BSA = 0.007184 × weight$^{0.425}$ × height$^{0.725}$) [43] and thus were accordingly presented, thus ensuring consistency in presentation without employing "index" nomenclature for clarity when discussion fundamental principles in Cardiovascular Physiology.

The blood pressure, which includes the systolic (SAP) and diastolic (DAP) arterial pressures, was assessed at rest in the upper left arm using the gold standard auscultatory method with a mercury sphygmomanometer and digital stethoscope equipped with sound amplification (3M™ Littmann CORE, USA). During exercise, blood pressure measurements were performed at a fixed relative high-intensity level [anaerobic threshold (AT)], determined by reaching a respiratory exchange ratio (RER) of 0.85 during the incremental exercise test [44]. Mean arterial pressure (MAP) was calculated taking into account the decreasing fraction of systole ($S_t = 0.01 \times e^{[4.14 - (40.74/HR)]}$) with increasing HR [MAP = (DAP + S_t) × pulse pressure] [45]. Systemic vascular resistance (SVR) was defined by the ratio of MAP and Q.

Aerobic capacity

VO_2, CO_2 output, and ventilation were continuously monitored using a mixing chamber system (KORR Medical, USA) during the incremental exercise test. Following a warm-up period at 10 – 30 W, the workload was gradually increased by 10 – 30 W increments every 50 s until exhaustion occurred within the recommended total duration of 7 – 9 min [46]. Prior to each test, calibration of the gas analyzers and the flowmeter was conducted. Values were averaged over a period of 15 s in accordance with current recommendations [47]. VO_{2peak}, defined as the highest average value, was determined if at least two of the following established criteria were met: (1) plateau in VO_2 despite increased workload, (2) HR_{peak} ± 10 bpm predicted based on age and body position [38,48], and/or (3) RER > 1

[49]. The arterio-venous difference in O_2 ($a\text{-}vO_{2diff}$) during incremental exercise was calculated by the Fick principle (VO_2 = LV Q × $a\text{-}vO_{2diff}$).

Intravascular volumes

The determination of red blood cell volume (RBCV), PV, and total BV was based on the primary measurement of circulating hemoglobin mass (Hb_{mass}) via the classic carbon monoxide (CO) rebreathing technique integrated in a semi-automated system with a low typical error of measurement (TE ≤ 1.2%), as previously detailed [34,50]. This validated method has been shown to accurately detect minor reductions in Hb_{mass} (–3%, 26 – 28 g) induced by phlebotomy in healthy young individuals [51]. Considering that Hb_{mass} is a tightly regulated variable that remains relatively stable for up to 2 weeks in healthy humans [52], CO-rebreathing was performed in the 1st testing visit (PBO); the obtained Hb_{mass} was used to determine intravascular volumes in both testing visits. Briefly, at rest (pre-infusion, before the exercise test), 2 ml blood was sampled from the cannulated antecubital vein and analyzed immediately in duplicate for percent carboxyhemoglobin (%HbCO), Hb concentration, and hematocrit (Hct; ABL80, Radiometer, USA) in both testing visits. Following the recovery period after exercise in the 1st visit, the participants breathed 100% O_2 along with a bolus of 1.5 ml/kg of 99.5% chemically pure CO (CO H.P. Grade, SG, Hong Kong, China) in a closed breathing circuit for 10 min. An additional sample of 2 ml blood was obtained exactly at the end of the 10-minute CO-rebreathing period and analyzed as aforementioned. The change in %HbCO induced by CO administration was used to calculate circulating Hb_{mass}, taking into account any residual amount of CO that remains in the rebreathing circuit after completion of the procedure. Hb_{mass}, along with resting pre-infusion Hb concentration and Hct levels, constitute essential variables required for determining RBCV, PV, and BV [34,50].

Body composition

Body composition was assessed via dual-energy X-ray absorptiometry (DXA; Hologic QDR 4500; Hologic, Inc., USA) according to current recommendations [53]. In brief, the participants were instructed to assume a seated position in the center of the scanning table and subsequently transitioned into a supine posture with their spine aligned with the printed longitudinal midline. Once in the supine position, their arms were placed alongside their body with the palms facing downwards to ensure a standardized scanning area for all individuals. Bone mineral content (BMC), lean body mass (LBM), and fat body mass were quantified.

Statistical analysis

Statistical analyses were performed using SPSS 26.0 (IBM). The normal distribution of the data was assessed using the Kolmogorov-Smirnov test, and the homogeneity of variances was tested with Levene’s test. The primary outcome was to determine the sex difference in the effect of intravenous

infusion (absolute delta change: Δ, BVexp minus PBO) on LV Q_{peak}. According to power analyses based on the unique precedent study assessing sex differences in the effect of BVexp on Q during exercise [17], a sample size of 20 participants provided 90% power to detect a 0.5 L/min higher Q (about +3% increment) at a fixed relative exercise intensity in men compared to women. Baseline variables and the effects of intravenous infusion at rest were compared between sexes via the independent *t*-test; within each sex, the dependent *t*-test was used to determine the effects of intravenous infusion at rest. During incremental exercise, two-way ANOVA with repeated measures was performed separately for women and men to assess the effect of the intravenous infusion on cardiac and pulmonary gas exchange variables. Condition (PBO, BVexp) and exercise intensity (60%, 70%, 80%, 90%, and 100% HR_{peak}) served as within-subject factors. To compare the effect of intravenous infusion (Δ, BVexp minus PBO) between sexes, a two-way ANOVA with repeated measures was employed considering sex (women, men) and exercise intensity (60%, 70%, 80%, 90%, and 100% HR_{peak}) as between- and within-subject factors, respectively. Post-hoc comparisons at each exercise intensity were conducted if the *F*-value obtained from ANOVA was significant using the Bonferroni correction method. A two-tailed *P*-value less than 0.05 was considered significant. All reported data were presented as mean ± SD.

Results

Baseline characteristics

All participants were non-smokers and had a healthy weight [body mass index (BMI) < 25 kg/m^2]. Anthropometrical measurements such as height, weight, BSA, as well as total BMC and LBM, both in absolute terms and relative to body weight, were lower in women compared to men ($P < 0.001$, **Additional file 1: Table S1**). Similarly, the resting blood O_2 carrying capacity of blood (Hb concentration) and Hct levels were lower in women ($P < 0.001$), whereas the total circulating BV per kilogram of body weight did not differ between genders ($P = 0.081$, **Additional file 1: Table S2**). Resting arterial blood pressures including SAP, DAP, and MAP were lower while SVR was higher in women than men ($P \leq 0.029$, **Additional file 1: Table S2**).

Effect of intravenous infusion on arterial blood pressures during exercise

The effects of PBO and BVexp on arterial blood pressures (SAP and MAP) at a fixed high-intensity exercise (AT) are displayed in **Additional file 1: Fig. S3**. Compared with PBO, BVexp did not significantly alter exercise arterial blood pressures in women and men ($P \geq 0.061$).

Discussion

Using a PBO-controlled, single-blinded, and cross-over design, this study invasively determined the isolated effect of increasing BV on cardiac and aerobic capacities in healthy young women and men

matched by age and physical activity. The main findings are as follows. (1) BVexp (+10%) induces nearly proportional increments in cardiac filling ($LVEDV_{peak}$) and LV SV_{peak}, regardless of sex. (2) The enhancing effect of BVexp on cardiac pumping capacity (LV Q_{peak}) is observed in women but attenuated in men due to concomitant decreases in HR_{peak}. (3) Both sexes experience a significant decrease in systemic O_2 extraction (a-$vO_{2diffpeak}$), along with reductions in SVR to blood flow during exercise (SVR_{AT}). And (4) VO_{2peak} is remarkably decreased with BVexp for both women and men.

The opening central finding supports a fundamental principle in cardiac physiology: a healthy young heart possesses an unexploited reserve to increase its filling capacity even during maximum exertion. Specifically, a moderate increase in BV leads to a corresponding increase in the filling of the primary and more stressed pumping chamber (LV). In contrast to the initial hypothesis, this cardiac distending effect was similarly observed in women and men at peak exercise. However, it should be noted that women exhibited lower levels of LV filling at rest and during submaximal exercise intensities compared to men (**Additional file 1: Table S2, Fig. S1**). Therefore, the hypothesized female-specific limitation in cardiac structure to accommodate additional BV may not be fully manifest until older age and/or the development of cardiovascular disease [24,25,30]. In healthy young women included in this study to eliminate the confounding factors such as age and/or disease, increased LV filling led to equivalent increases in LV SV and Q_{peak} via the Frank-Starling mechanism (**Additional file 1: Fig. S1**). Thus, interventions exclusively targeting BV can potentially enhance cardiac capacity for women during young adulthood. Surprisingly, although the Frank-Starling mechanism remained intact in men with augmented peak LV filling and LV SV due to BVexp, Q_{peak} did not proportionally increase due to reduced HR_{peak} (**Additional file 1: Fig. S1**). The fact that markers of maximal volitional effort such as the plateau in O_2 uptake and peak RER remained unchanged under the experimental condition implies the presence of a true negative chronotropic effect of BVexp in men. The specific reduction (–5%) in HR_{peak} induced by BVexp in this study was previously reported after 8-week endurance training leading to increased BV (+8%) in healthy young men [54,55]. Intriguingly, this decrease in HR_{peak} was only observed after upright endurance training and when tested in the supine position. In a parallel group assigned to supine endurance training where BV was not increased but LV filling, LV SV, and Q during exercise were markedly enhanced, HR_{peak} remained preserved [54,55]. Nonetheless, the mechanisms underlying the acute response of HR_{peak} to BVexp observed in this study may differ from those explaining similar effects with (chronic) endurance training. It is possible that specific hemodynamic stimuli targeting neural (autonomic) responses/adaptations contributing to the regulation of HR are required for men to fully benefit from increased cardiac filling. This point emphasizes the need for additional responses/adaptations other than hypervolemia to enhance cardiac and aerobic capacities, and will be further discussed.

Arterial blood pressure is a tightly regulated variable in the circulatory system. As demonstrated during rest, both SAP and DAP were stable despite a significant increase in LV Q due to BVexp in women and men (**Additional file 1: Table S2**). This homeostatic regulation can be attributed to an equal reduction of SVR in both sexes (**Additional file 1: Table S2**). Consequently, when the filling of the heart and subsequent Q are acutely increased via the Frank-Starling mechanism, compensatory vasodilation ensures that arterial blood pressure remains constant in healthy individuals at rest. During exercise involving the activation of more than 50% of total muscle mass (e.g., leg cycling), there is commonly a marked decrease in SVR, which facilitates an increase in LV Q for a given arterial blood pressure in women and men [34,35,56]. As demonstrated by Calbet et al. [56], an optimal reduction of SVR occurs at peak effort where supraphysiological stimuli such as exogenous vasodilating drugs further decrease SVR while increasing Q_{peak} at the expense of inefficient blood flow distribution. Specifically, the intraarterial (femoral) infusion of ATP, a potent vasodilator, during peak cycling exercise reduces SVR without altering arterial blood pressure, leading to an increased Q_{peak} along with proportional decremental changes in systemic O_2 extraction (a-$vO_{2diffpeak}$) [56]. Given that O_2 delivery limits O_2 consumption capacity in humans [57], supplementary ATP-induced peripheral vasodilation was elicited in, and the secondary increase in Q_{peak} was not directed towards, the active (high O_2-consuming) muscle fibers [56]. A similar peripheral outcome can be prompted by BVexp. As displayed in **Additional file 1: Fig. S3**, BVexp exerts its greatest impact on a-vO_{2diff} during incremental exercise, characterized by significant reductions in SVR but no change in arterial blood pressure, regardless of sex. It can be inferred that during incremental exercise either 1) arterial blood pressure reaches its maximum and the hemodynamic stress on the circulatory system (represented by the product of Q and SVR) becomes the limiting factor, leading to a reduction in SVR (in an inefficient manner) proportional to the BVexp-induced increase in Q, or 2) it is strictly regulated up to a certain point [58]. The former hypothesis can be discarded as arterial blood pressure is not maximal during incremental cycling exercise in healthy young individuals [59]. Moreover, improvements in cardiac and aerobic capacities in healthy humans through endurance training are associated with significant increases in arterial blood pressure during incremental exercise [60,61]. Furthermore, it should be noted that decreases in blood O_2 carrying capacity alone without altering BV do not affect Q and SVR during incremental exercise, suggesting that the circulatory effects induced by BVexp are not primarily mediated by local O_2-dependent mechanisms of peripheral vasodilation [62]. Taken together, complementary responses/adaptations modifying the systemic regulation of arterial blood pressure must coexist with hypervolemia in order that enhanced Q and O_2-rich arterial blood can be directed towards the vessels supplying working muscles of women and men.

Aerobic capacity, also known as VO_{2peak}, represents the maximum rate of O_2 transport and utilization in the body's circulatory system. It serves as a reliable indicator of both clinical fitness and

endurance performance [7]. Over the course of more than six decades, extensive efforts have been made to understand the factors influencing VO_{2peak} and identify effective strategies for its enhancement [63]. Our research, along with others, has attributed a significant role to BV, supported by consistent negative effects observed when BV is reduced through blood withdrawal [3,4,34]. Notably, training-induced improvements in VO_{2peak} are abolished when gains in BV are negated [64-66]. However, it remains unclear whether the relationship between BV and VO_{2peak} established under "negative" conditions can be directly extrapolated to the "positive" side due to uncontrolled factors present in previous investigations on BVexp explained in the "Background" section [2,5,12-20]. Another point worth discussing is the notable discrepancy between the effects of BVexp on VO_{2peak} and endurance performance, with the latter being defined as time to exhaustion during incremental or high-intensity (approximately 95% HR_{peak}) exercise [2,5,12,15,17]. While BVexp generally had no impact on VO_{2peak}, there was a significant decrease in endurance performance (up to a –20% reduction) observed in most studies [2,5,12,15,17]. The majority of these studies reported exacerbated fatigue in the lower limb along with reduced endurance performance [2,5,12,15,17]. It is possible that the excessive plasma leakage from circulation into the interstitial fluid of the lower limb (due to elevated hydrostatic pressure caused by BVexp in an upright body position) contributed to increased peripheral fatigue and impaired performance [67], while having a relatively lesser negative effect on VO_{2peak} [2,5,12,15,17]. The present study indicates that when the body position is controlled to minimize hydrostatic pressure and thereby preserve the infusate within the circulation, both VO_{2peak} and endurance performance are significantly impaired, as expected due to their strong established association, in women and men. Of note, these investigated effects were acute in nature raising questions about timing considerations. Until further evidence becomes available, it cannot be ruled out that prolonged BVexp itself eventually stimulates adaptations in baroregulation that optimize peripheral blood flow and enhance aerobic energy production even without exercise training.

The cardiac assessment was performed non-invasively using echocardiography, which may involve a moderate margin of measurement error (6%) and an underestimation of LV volumes [34,35]. However, due to its high temporal resolution, echocardiography is necessary for imaging the heart during high-intensity exercise. It should be noted that this study has the largest sample size on this topic, enhancing the statistical power in relation to previous studies for primary and secondary outcomes. Additionally, the participants were blinded to the type of intravenous infusion administered. Nevertheless, it remains unclear how the basal degree of circulatory system filling could potentially influence the effect of the intravenous infusion on cardiovascular function. Further research is needed to establish what constitutes "normal" BV and circulatory filling in humans [68]. Despite this uncertainty, BV per kilogram of body weight was matched between sexes, and RA volumes (indicative of overall fluid status) corresponded with normative values according to sex, age, and ethnicity [69].

Likewise, the average hemodilution induced by albumin was identical in women and men (17%), suggesting that PV distribution within the circulatory system did not confound the sex comparison. Finally, blood pressure was not assessed at peak effort but rather close to it; previous studies using the same exercise protocol have shown a plateau in SAP, DAP, and MAP among healthy individuals [3,34].

References

1. Kjellberg SR, Rudhe U, Sjöstrand T. Increase of the amount of hemoglobin and blood volume in connection with physical training. Acta Physiol Scand. 1949;19(2-3):146-51.
2. Warburton DE, Gledhill N, Jamnik VK, Krip B, Card N. Induced hypervolemia, cardiac function, VO_{2max}, and performance of elite cyclists. Med Sci Sports Exerc. 1999;31(6):800-8.
3. Diaz-Canestro C, Pentz B, Sehgal A, Montero D. Differences in cardiac output and aerobic capacity between sexes are explained by blood volume and oxygen carrying capacity. Front Physiol. 2022;13:747903.
4. Diaz-Canestro C, Pentz B, Sehgal A, Montero D. Blood withdrawal acutely impairs cardiac filling, output and aerobic capacity in proportion to induced hypovolemia in middle-aged and older women. Appl Physiol Nutr Metab. 2021.
5. Kanstrup IL, Ekblom B. Acute hypervolemia, cardiac performance, and aerobic power during exercise. J Appl Physiol Respir Environ Exerc Physiol. 1982;52(5):1186-91.
6. Lundby C, Montero D, Joyner M. Biology of VO_2 max: looking under the physiology lamp. Acta Physiol (Oxf). 2017;220(2):218-28.
7. Kodama S, Saito K, Tanaka S, Maki M, Yachi Y, Asumi M, et al. Cardiorespiratory fitness as a quantitative predictor of all-cause mortality and cardiovascular events in healthy men and women: a meta-analysis. JAMA. 2009;301(19):2024-35.
8. Lundby C, Robach P, Saltin B. The evolving science of detection of 'blood doping'. Br J Pharmacol. 2012;165(5):1306-15.
9. Ekblom B, Goldbarg AN, Gullbring B. Response to exercise after blood loss and reinfusion. J Appl Physiol. 1972;33(2):175-80.
10. Chomsky DB, Lang CC, Rayos GH, Shyr Y, Yeoh TK, Pierson RN, 3rd, et al. Hemodynamic exercise testing. A valuable tool in the selection of cardiac transplantation candidates. Circulation. 1996;94(12):3176-83.
11. Metra M, Faggiano P, D'aloia A, Nodari S, Gualeni A, Raccagni D, et al. Use of cardiopulmonary exercise testing with hemodynamic monitoring in the prognostic assessment of ambulatory patients with chronic heart failure. J Am Coll Cardiol. 1999;33(4):943-50.
12. Coyle EF, Hemmert MK, Coggan AR. Effects of detraining on cardiovascular responses to exercise: role of blood volume. J Appl Physiol (1985). 1986;60(1):95-9.
13. Coyle EF, Hopper MK, Coggan AR. Maximal oxygen uptake relative to plasma volume expansion. Int J Sports Med. 1990;11(2):116-9.
14. Hopper MK, Coggan AR, Coyle EF. Exercise stroke volume relative to plasma-volume expansion. J Appl Physiol (1985). 1988;64(1):404-8.

15. Kanstrup IL, Ekblom B. Blood volume and hemoglobin concentration as determinants of maximal aerobic power. Med Sci Sports Exerc. 1984;16(3):256-62.
16. Krip B, Gledhill N, Jamnik V, Warburton D. Effect of alterations in blood volume on cardiac function during maximal exercise. Med Sci Sports Exerc. 1997;29(11):1469-76.
17. Mier CM, Domenick MA, Turner NS, Wilmore JH. Changes in stroke volume and maximal aerobic capacity with increased blood volume in men women. J Appl Physiol (1985). 1996;80(4):1180-6.
18. Berger NJ, Campbell IT, Wilkerson DP, Jones AM. Influence of acute plasma volume expansion on VO_2 kinetics, VO_{2peak}, and performance during high-intensity cycle exercise. J Appl Physiol (1985). 2006;101(3):707-14.
19. Zavorsky GS, Walley KR, Hunte GS, Mckenzie DC, Sexsmith GP, Russell JA. Acute hypervolemia lengthens red cell pulmonary transit time during exercise in endurance athletes. Respir Physiol Neurobiol. 2002;131(3):255-68.
20. Zavorsky GS, Walley KR, Hunte GS, Mckenzie DC, Sexsmith GP, Russell JA. Acute hypervolaemia improves arterial oxygen pressure in athletes with exercise-induced hypoxaemia. Exp Physiol. 2003;88(4):555-64.
21. Lundvall J, Bjerkhoel P. Failure of hemoconcentration during standing to reveal plasma volume decline induced in the erect posture. J Appl Physiol (1985). 1994;77(5):2155-62.
22. Lundvall J, Bjerkhoel P, Quittenbaum S, Lindgren P. Rapid plasma volume decline upon quiet standing reflects large filtration capacity in dependent limbs. Acta Physiol Scand. 1996;158(2):161-7.
23. Sheel AW, Dominelli PB, Molgat-Seon Y. Revisiting dysanapsis: sex-based differences in airways and the mechanics of breathing during exercise. Exp Physiol. 2016;101(2):213-8.
24. Regitz-Zagrosek V, Kararigas G. Mechanistic pathways of sex differences in cardiovascular disease. Physiol Rev. 2017;97(1):1-37.
25. Diaz-Canestro C, Montero D. Sex and age interaction in fundamental circulatory volumetric variables at peak working capacity. Biol Sex Differ. 2022;13(1):1.
26. Diaz-Canestro C, Pentz B, Sehgal A, Montero D. Sex dimorphism in cardiac and aerobic capacities: the influence of body composition. Obesity (Silver Spring). 2021;29(11):1749-59.
27. Diaz-Canestro C, Montero D. The impact of sex on left ventricular cardiac adaptations to endurance training: a systematic review and meta-analysis. Sports Med. 2020;50(8):1501-13.
28. Howden EJ, Perhonen M, Peshock RM, Zhang R, Arbab-Zadeh A, Adams-Huet B, et al. Females have a blunted cardiovascular response to one year of intensive supervised endurance training. J Appl Physiol (1985). 2015;119(1):37-46.

29. Montero D, Breenfeldt-Andersen A, Oberholzer L, Haider T, Goetze JP, Meinild-Lundby AK, et al. Erythropoiesis with endurance training: dynamics and mechanisms. Am J Physiol Regul Integr Comp Physiol. 2017;312(6):R894-R902.
30. Redfield MM, Jacobsen SJ, Borlaug BA, Rodeheffer RJ, Kass DA. Age- and gender-related ventricular-vascular stiffening: a community-based study. Circulation. 2005;112(15):2254-62.
31. Montero D, Houben AJ, Koster A, Muris DM, Schram MT, Gronenschild EH, et al. Physical activity is associated with glucose tolerance independent of microvascular function: the maastricht study. J Clin Endocrinol Metab. 2016;101(9):3324-32.
32. Montero D, Dandanell S, Oberholzer L, Keiser S, Breenfeldt-Andersen A, Haider T, et al. Combined effects of physical inactivity and acute hyperglycemia on arterial distensibility. Vasc Med. 2017;22(4):285-91.
33. Keller MF, Harrison ML, Lalande S. Impact of menstrual blood loss and oral contraceptive use on oxygen-carrying capacity. Med Sci Sports Exerc. 2020;52(6):1414-9.
34. Diaz-Canestro C, Pentz B, Sehgal A, Montero D. Sex differences in cardiorespiratory fitness are explained by blood volume and oxygen carrying capacity. Cardiovasc Res. 2022;118(1):334-43.
35. Diaz-Canestro C, Pentz B, Sehgal A, Yang R, Xu A, Montero D. Lean body mass and the cardiovascular system constitute a female-specific relationship. Sci Transl Med. 2022;14(667):eabo2641.
36. Boda WL, Watenpaugh DE, Ballard RE, Hargens AR. Supine lower body negative pressure exercise simulates metabolic and kinetic features of upright exercise. J Appl Physiol (1985). 2000;89(2):649-54.
37. Eiken O. Effects of increased muscle perfusion pressure on responses to dynamic leg exercise in man. Eur J Appl Physiol Occup Physiol. 1988;57(6):772-6.
38. Dillon HT, Dausin C, Claessen G, Lindqvist A, Mitchell A, Wright L, et al. The effect of posture on maximal oxygen uptake in active healthy individuals. Eur J Appl Physiol. 2021;121(5):1487-98.
39. Guo M, Diaz-Canestro C, Ng MY, Yiu KH, Montero D. Sex-specific limitation of cardiac capacity during the adult life span: return to fundamental structure and function. J Gerontol A Biol Sci Med Sci. 2024;79(3).
40. Lang RM, Badano LP, Mor-Avi V, Afilalo J, Armstrong A, Ernande L, et al. Recommendations for cardiac chamber quantification by echocardiography in adults: an update from the American Society of Echocardiography and the European Association of Cardiovascular Imaging. Eur Heart J Cardiovasc Imaging. 2015;16(3):233-70.

41. Pellikka PA, Nagueh SF, Elhendy AA, Kuehl CA, Sawada SG, American Society of Echocardiography. American Society of Echocardiography recommendations for performance, interpretation, and application of stress echocardiography. J Am Soc Echocardiogr. 2007;20(9):1021-41.
42. Mertens LL, Friedberg MK. Imaging the right ventricle--current state of the art. Nat Rev Cardiol. 2010;7(10):551-63.
43. Du Bois D, Du Bois EF. A formula to estimate the approximate surface area if height and weight be known. Nutrition. 1989;5(5):303-11; discussion 12-3.
44. Wiecha S, Kasiak PS, Cieśliński I, Maciejczyk M, Mamcarz A, Śliż D. Modeling physiological predictors of running velocity for endurance athletes. J Clin Med. 2022;11(22):6688.
45. Moran D, Epstein Y, Keren G, Laor A, Sherez J, Shapiro Y. Calculation of mean arterial pressure during exercise as a function of heart rate. Appl Human Sci. 1995;14(6):293-5.
46. Astorino TA, Rietschel JC, Tam PA, Taylor K, Johnson SM, Freedman TP, et al. Reinvestigation of optimal duration of VO_{2max} testing. J Exerc Physiology. 2004;7(6):1-8.
47. Martin-Rincon M, Calbet JAL. Progress update and challenges on VO_{2max} testing and interpretation. Front Physiol. 2020;11:1070.
48. Nes BM, Janszky I, Wisloff U, Stoylen A, Karlsen T. Age-predicted maximal heart rate in healthy subjects: the HUNT fitness study. Scand J Med Sci Sports. 2013;23(6):697-704.
49. American Thoracic Society, American College of Chest Physicians. ATS/ACCP statement on cardiopulmonary exercise testing. Am J Respir Crit Care Med. 2003;167(2):211-77.
50. Siebenmann C, Keiser S, Robach P, Lundby C. CORP: the assessment of total hemoglobin mass by carbon monoxide rebreathing. J Appl Physiol (1985). 2017;123(3):645-54.
51. Keiser S, Meinild-Lundby AK, Steiner T, Trosch S, Rauber S, Krafft A, et al. Detection of blood volumes and haemoglobin mass by means of CO re-breathing and indocyanine green and sodium fluorescein injections. Scand J Clin Lab Invest. 2017;77(3):164-74.
52. Montero D, Lundby C. Regulation of red blood cell volume with exercise training. Compr Physiol. 2018;9(1):149-64.
53. Lewiecki EM, Binkley N, Morgan SL, Shuhart CR, Camargos BM, Carey JJ, et al. Best practices for dual-energy X-ray absorptiometry measurement and reporting: International Society for clinical densitometry guidance. J Clin Densitom. 2016;19(2):127-40.
54. Ray CA, Cureton KJ. Interactive effects of body posture and exercise training on maximal oxygen uptake. J Appl Physiol (1985). 1991;71(2):596-600.
55. Ray CA, Cureton KJ, Ouzts HG. Postural specificity of cardiovascular adaptations to exercise training. J Appl Physiol (1985). 1990;69(6):2202-8.

56. Calbet JA, Lundby C, Sander M, Robach P, Saltin B, Boushel R. Effects of ATP-induced leg vasodilation on VO_2 peak and leg O_2 extraction during maximal exercise in humans. Am J Physiol Regul Integr Comp Physiol. 2006;291(2):R447-53.
57. Boushel R, Gnaiger E, Calbet JA, Gonzalez-Alonso J, Wright-Paradis C, Sondergaard H, et al. Muscle mitochondrial capacity exceeds maximal oxygen delivery in humans. Mitochondrion. 2011;11(2):303-7.
58. Walgenbach SC, Donald DE. Inhibition by carotid baroreflex of exercise-induced increases in arterial pressure. Circ Res. 1983;52(3):253-62.
59. Brink-Elfegoun T, Kaijser L, Gustafsson T, Ekblom B. Maximal oxygen uptake is not limited by a central nervous system governor. J Appl Physiol (1985). 2007;102(2):781-6.
60. Ekblom B, Astrand PO, Saltin B, Stenberg J, Wallstrom B. Effect of training on circulatory response to exercise. J Appl Physiol. 1968;24(4):518-28.
61. Tanaka H, Bassett DR Jr, Turner MJ. Exaggerated blood pressure response to maximal exercise in endurance-trained individuals. Am J Hypertens. 1996;9(11):1099-103.
62. Diaz-Canestro C, Siebenmann C, Montero D. Blood oxygen carrying capacity determines cardiorespiratory fitness in middle-age and older women and men. Med Sci Sports Exerc. 2021;53(11):2274-82.
63. Mitchell JH, Sproule BJ, Chapman CB. The physiological meaning of the maximal oxygen intake test. J Clin Invest. 1958;37(4):538-47.
64. Bonne TC, Doucende G, Fluck D, Jacobs RA, Nordsborg NB, Robach P, et al. Phlebotomy eliminates the maximal cardiac output response to six weeks of exercise training. Am J Physiol Regul Integr Comp Physiol. 2014;306(10):R752-R60.
65. Montero D, Cathomen A, Jacobs RA, Fluck D, De Leur J, Keiser S, et al. Haematological rather than skeletal muscle adaptations contribute to the increase in peak oxygen uptake induced by moderate endurance training. J Physiol. 2015;593(20):4677-88.
66. Mandic M, Eriksson LMJ, Melin M, Skott V, Sundblad P, Gustafsson T, et al. Increased maximal oxygen uptake after sprint-interval training is mediated by central haemodynamic factors as determined by right heart catheterization. J Physiol. 2023;601(12):2359-70.
67. Antle DM, Cormier L, Findlay M, Miller LL, Cote JN. Lower limb blood flow and mean arterial pressure during standing and seated work: Implications for workplace posture recommendations. Prev Med Rep. 2018;10:117-22.
68. Feldschuh J, Enson Y. Prediction of the normal blood volume. Relation of blood volume to body habitus. Circulation. 1977;56(4 Pt 1):605-12.

69. Soulat-Dufour L, Addetia K, Miyoshi T, Citro R, Daimon M, Fajardo PG, et al. Normal values of right atrial size and function according to age, sex, and ethnicity: results of the World Alliance Societies of Echocardiography Study. J Am Soc Echocardiogr. 2021;34(3):286-300.
